# Can Time Determine Preanalytical Quality? A Temporal Analysis of Specimen Rejection Rates

**DOI:** 10.3390/jcm15124752

**Published:** 2026-06-18

**Authors:** Bağnu Dündar, Betül Özbek, Fatma Bozkurt, Asiye Gok Yurttas

**Affiliations:** 1 Department of Medical Biochemistry, Faculty of Medicine, Istanbul Atlas University, 34403 Istanbul, Türkiye; bagnu.dundar@atlas.edu.tr (B.D.); betul.ozbek@atlas.edu.tr (B.Ö.); 2 Department of Infectious Diseases and Clinical Microbiology, Faculty of Medicine, Istanbul Atlas University, 34403 Istanbul, Türkiye; fatma.bozkurt@atlas.edu.tr

**Keywords:** preanalytical phase, specimen rejection, laboratory errors, time factor, quality indicators, hemolysis, patient safety

## Abstract

**Objective:** Preanalytical errors account for the vast majority of preanalytical incidents and remain a fundamental threat to the reliability of test results. Although the types and frequencies of these errors have been extensively studied in the literature, their time-dependent variability has received comparatively little attention. This study aimed to evaluate how preanalytical specimen rejection rates vary across intraday time intervals and to assess the independent influence of time on preanalytical quality. **Methods:** This retrospective observational study included a total of 579,845 specimens accepted by the central laboratory of Istanbul Atlas University Hospital between January 2024 and December 2025. Specimens were analyzed with respect to preanalytical rejection reasons, the distribution and rate of these reasons across clinical units, and time of day. Each day was divided into six equal four-hour intervals: Z1 (00:00–04:00), Z2 (04:00–08:00), Z3 (08:00–12:00), Z4 (12:00–16:00), Z5 (16:00–20:00), and Z6 (20:00–24:00). Statistical analyses were performed using the Pearson chi-square test, and effect sizes were quantified using Cramér’s V coefficient. **Results:** Of the 579,845 specimens examined, 4365 were rejected, yielding an overall rejection rate of 0.79%. Rejection rates were found to be non-uniformly distributed across the day (*p* < 0.001). The highest rejection rate was observed during the Z2 interval (04:00–08:00) at 1.98%, whereas the lowest was recorded during Z3 (08:00–12:00) at 0.45%. Negative binomial regression analysis identified the Z2 interval as the only time period independently associated with an increased rejection risk Incidence Rate Ratio (IRR) = 1.63; 95% Confidence Interval (CI): 1.22–2.19. Among clinical units, the highest rejection rate was recorded in the emergency department (1.92%). Analysis of error types revealed that the majority of rejections were attributable to hemolysis (47.5%) and clotted specimens (26.3%). Hemolysis rates peaked in the emergency department, while clotted specimens occurred more frequently within intensive care units. Analysis of time and error interactions revealed that clotted specimens peaked during Z1 and Z2, whereas hemolysis became the primary cause of rejection during Z3 and Z4. **Conclusions:** Preanalytical specimen rejection rates exhibited significant variation according to time of day, clinical unit, and error type, with time emerging as a factor independently associated with preanalytical quality. The coexistence of elevated rejection risk during Z2 (04:00–08:00) and markedly low rejection rates during Z3 (08:00–12:00) indicates that the relationship between workload and error frequency is not linear. Although hemolysis and clotted specimens constituted the dominant error types, their distribution followed distinct patterns depending on the clinical unit and time interval. These results underscore the necessity of time-based monitoring to pinpoint unit-specific risks, providing a clear roadmap for targeted quality improvement interventions.

## 1. Introduction

Quality in laboratory medicine is defined not solely by the accuracy of test results, but also by the conditions under which those results are obtained. Although the total testing process encompasses preanalytical, analytical, and postanalytical phases, evidence clearly demonstrates that the overwhelming majority of laboratory errors arise outside the analytical phase, particularly during the preanalytical stage [1,2,3]. This renders the preanalytical phase the most critical and inherently variable component of the laboratory quality continuum.

The preanalytical phase encompasses a multitude of interdependent variables, including specimen collection, transportation, and preparation for analysis. Among these, hemolysis, clot formation, and insufficient specimen volume represent the most frequently encountered sources of error [4,5,6]. The occurrence of such errors is substantially shaped by human factors, workload dynamics, organizational processes, and technical practices, all of which interact in ways that are difficult to standardize across diverse clinical settings [7,8].

A defining characteristic of these variables is their temporal dependency. Patient admission rates, specimen reception volumes, and staffing distributions exhibit pronounced fluctuations throughout the day. Despite this well-recognized variability, prevailing quality indicators in laboratory medicine continue to evaluate preanalytical performance largely independent of temporal context [9,10,11]. Although the significance of human factors and workload has received increasing attention in recent years, these determinants are themselves inherently time-dependent phenomena, a dimension that remains insufficiently addressed in the existing literature [12,13].

In this context, time may be conceptualized not merely as one component of the preanalytical process, but as a higher-order parameter that integrates and reflects the cumulative influence of all other contributing variables. The present study analyzed the distribution of preanalytical error rates across distinct intraday time intervals, with the aim of investigating whether time can serve as a meaningful indicator of preanalytical quality.

## 2. Materials and Methods

### 2.1. Study Design and Data Collection

This study was conducted at the Central Laboratory of Istanbul Atlas University Hospital, a tertiary-care academic medical center serving both inpatient and outpatient populations. According to institutional records for the 2024–2025 period, a total of 692,680 patients presented to the hospital. During the same two-year period, laboratory testing was requested for 320,047 patients, and a total of 579,845 specimens were processed in the central laboratory. Specimens are received from multiple clinical departments, including intensive care units, the emergency department, clinical units, outpatient clinics, and surgical units. Routine samples are primarily transported to the central laboratory through a standardized pneumatic tube system, while critical or time-sensitive specimens are transferred directly by trained healthcare personnel to minimize preanalytical delays and preserve sample integrity. The laboratory performs a broad range of diagnostic tests, including routine clinical chemistry, hematology, coagulation, immunoassay, and hormone analyses.

Data recorded in the Laboratory Information System (LIS) of this institution between 1 January 2024 and 31 December 2025 were retrospectively analyzed. Information regarding preanalytical rejection reasons, the submitting clinical unit, and the timing of rejection processing was extracted from the LIS and included in the analysis. The dataset was retrieved without patient-level identifiable information or clinical variables such as patient identity, age, and diagnosis. Data extraction was performed in collaboration with qualified institutional LIS specialists; custom query codes were developed and the extraction process was iteratively validated by the technical personnel responsible for system management to ensure accuracy and completeness. The study was conducted in accordance with the Declaration of Helsinki and received approval from the Institutional Ethics Committee (Istanbul Atlas University Non-Interventional Scientific Research Ethics Committee, Decision No. 02/97, dated 23 February 2026).

### 2.2. Temporal Framework and Clinical Unit Classification

The 24 h sampling period was divided into six equal four-hour intervals (00:00–04:00 (Z1), 04:00–08:00 (Z2), 08:00–12:00 (Z3), 12:00–16:00 (Z4), 16:00–20:00 (Z5), and 20:00–24:00 (Z6)) in accordance with the healthcare quality standards published by the Turkish Ministry of Health, which mandate the evaluation of preanalytical processes across six standardized time windows as a national laboratory quality indicator. This temporal assessment framework is designed to facilitate institutional quality monitoring, workforce optimization, and operational workload management by enabling the evaluation of time-dependent variations in laboratory performance indicators. On methodological grounds, equal interval widths ensure balanced sample accumulation across all time periods, allow for the detection of clinically meaningful temporal variation, and minimize bias that may arise from unequal groupings. Additionally, this framework enabled the identification of hospital units with the highest error rates, providing an evidence-based foundation for decisions regarding the retention and deployment of trained personnel for patient safety. The clinical units from which specimens were submitted were classified into four main categories: outpatient clinics, clinical units, the emergency department, and intensive care units.

### 2.3. Rejection Reason Classification and Rate Calculation

Rejection reasons were examined based on the categories defined in the LIS in accordance with the Ministry of Health specimen acceptance and rejection criteria, and included hemolysis, clot formation, insufficient specimen volume, inappropriate specimen collection, incorrect container or tube selection, repeated specimen collection, empty specimen container, inappropriate transfer, exceeding the maximum allowable transfer time, erroneous patient identification, incorrect barcoding, specimen collected from the wrong patient, lipemia, icterus, and failure to deliver the specimen to the laboratory.

The overall rejection rate was calculated by dividing the total number of rejected specimens by the total number of specimens received. Unit- and time interval-based rejection rates were computed using the same approach. Specimens were categorized into four types: urine, whole blood, serum, and plasma. Other specimen types received by the laboratory, including stool, body fluid, semen analysis, and microbiological culture specimens, were not included in the study. Rejection reasons were subsequently analyzed according to specimen type. For hemolyzed specimens, serum and plasma served as the denominator; for clotted specimens, whole blood and plasma were used; and for all other rejection reasons, the total specimen count was applied as the denominator. Unit- and time interval-specific rejection rates were calculated by dividing the number of rejections recorded within a given unit and time interval by the total number of relevant specimens received within that same unit and time interval.

Sample quality assessment was performed according to the routine preanalytical workflow. Upon receipt, all specimens were visually evaluated by trained laboratory technicians at the sample acceptance station for visible hemolysis and clot formation. Samples demonstrating macroscopically detectable hemolysis or clotting were rejected prior to analysis. Samples accepted for analysis were additionally monitored using analyzer-integrated clot detection systems, and samples flagged as clotted were excluded. Hemolysis assessment for serum samples was performed using serum indices (HIL index methodology). In routine practice, samples identified as hemolyzed through HIL assessment were not automatically rejected; instead, interpretative comments were added to laboratory reports and repeat sampling was recommended when appropriate. The present study evaluated only rejections occurring during the preanalytical phase.

### 2.4. Sample Transport Logistics and Quality Control Mechanisms

The study was conducted in a centralized teaching and research hospital operating on a single-site campus without geographically separate or remote satellite clinics. The primary outpatient blood collection units are located in immediate proximity to the central laboratory. For clinical units, intensive care units, and the emergency department, specimen transport is facilitated via an automated pneumatic tube system, ensuring delivery to the laboratory reception within minutes of collection. To prevent preanalytical transport delays and ensure sample integrity, a tri-level verification protocol was enforced within the 24 h cycle. Trained laboratory personnel and specialists cross-reference system records with physical arrivals at three scheduled daily checkpoints: the morning check (during the Z3 interval), the afternoon check (during the Z4 interval), and the night shift check (across the Z1/Z6 intervals) to confirm no specimens remain pending in the pneumatic lines.

Specimen acceptance and rejection rules were governed by standardized national criteria defined by the Turkish Ministry of Health. Rejection criteria were strictly monitored and logged through the LIS. Hemolysis and lipemia/icterus were verified through visual inspection or automated serum indices. Clot formation was assessed via physical inspection with applicators or during routine pre-centrifugation checks. Volumetric errors (insufficient or inappropriate sample volume) were determined based on the exact fill-lines marked on specific evacuated tube systems. Identification and barcoding errors were flagged at the reception point by comparing LIS electronic orders against physical tube labels before processing.

### 2.5. Data Stratification and Temporal Analysis:

To facilitate a robust temporal analysis of preanalytical error distribution, the 24 h cycle was systematically stratified into six equal four-hour intervals (00:00–04:00, 04:00–08:00, 08:00–12:00, 12:00–16:00, 16:00–20:00, and 20:00–24:00). The selection of four-hour increments was a deliberate methodological choice designed to optimize the trade-off between temporal granularity and statistical power. Broad temporal grouping (e.g., 8 h shift-based analysis) carries an inherent risk of masking transient, shift-dependent variations in error rates that are often diluted in larger datasets. Conversely, excessively narrow intervals (e.g., 1 h segments) frequently result in low event counts, thereby compromising the statistical significance of the findings. By adopting four-hour intervals, we achieved a granular resolution sufficient to capture clinically meaningful deviations in preanalytical performance—such as those occurring during peak operational volumes or handover periods—while maintaining a sample density adequate for reliable statistical inference. This standardized framework ensures comparability across analytical units and minimizes potential biases arising from unequal temporal grouping, aligning our study design with established best practices in preanalytical clinical laboratory research.

### 2.6. Statistical Analysis

Statistical analyses were performed using IBM SPSS Statistics for Windows, version 27.0 (IBM Corp., Armonk, NY, USA). Pearson’s chi-square test was used to compare the distributions of specimen rejection events across clinical units, time intervals, and rejection categories. Statistical significance was defined as *p* < 0.05. For comparisons yielding statistically significant results, Cramér’s V coefficient was calculated to quantify effect size, and adjusted standardized residuals were examined to identify the specific category combinations contributing to the observed differences. Because several rejection reasons occurred infrequently within certain clinical-unit and time-interval subgroups, resulting in sparse contingency table cells and potentially unreliable statistical estimates, individual rejection causes were consolidated into five major categories: hemolysis, clot formation, insufficient specimen volume, inappropriately collected specimen or incorrect container/tube, and other. The “other” category included repeated specimen collection, empty specimen containers, inappropriate specimen transfer, specimens exceeding the maximum allowable transport time, patient identification errors, incorrect barcoding, specimens collected from the wrong patient, lipemia, icterus, and specimens not delivered to the laboratory. Although absolute rejection counts were initially evaluated for descriptive purposes, rejection rates were prioritized for comparative analyses because they provide a more accurate assessment of specimen quality by accounting for differences in specimen volume among clinical units and sampling periods. Reliance solely on raw rejection counts may lead to misleading conclusions when substantial variation exists in the number of submitted specimens; therefore, all primary comparisons were based on normalized rejection rates. To further improve the precision and interpretability of rejection rate calculations and incidence rate ratio (IRR) estimates, a tube-specific normalization strategy was applied. Rather than using the total specimen volume as a universal denominator for all rejection categories, denominators were adjusted according to the biological and technical relevance of each specimen type. Hematological specimens were excluded from hemolysis-related rejection rate calculations because hemolysis is not considered a routine rejection criterion for these specimens under institutional laboratory procedures. Additionally, tube-type information was unavailable in our dataset; therefore, specimen numbers and rejection rates were determined directly according to sample type. This approach minimized potential confounding arising from differences in test requirements among specimen types and allowed rejection rates to more accurately reflect the risk profile associated with clinically relevant sample categories.

A negative binomial regression model was subsequently constructed to estimate the relative risk of specimen rejection. The outpatient clinic and the Z3 time interval, which demonstrated the lowest rejection rates, were designated as the reference categories. Negative binomial regression was preferred over a Poisson model because the distribution of rejection events exhibited overdispersion and substantial variability across groups. The corresponding specimen volume for each rejection category was incorporated into the model as an offset variable. Results are presented as incidence rate ratios (IRRs) with 95% confidence intervals and associated *p*-values. An IRR greater than 1 indicates an increased relative risk of specimen rejection compared with the reference category, whereas an IRR less than 1 indicates a reduced relative risk.

## 3. Results

Of the 552,450 specimens (n = 316,243 in 2024, n = 236,207 in 2025) accepted by the central laboratory during the 2024–2025 study period, 4365 (n = 2221 in 2024, n = 2144 in 2025) were rejected for preanalytical reasons, yielding an overall rejection rate of 0.79% (0.7% in 2024, 0.9% in 2025).

### 3.1. Rejection Counts and Rates by Units

When rejection counts were evaluated at the unit level, the highest absolute number of rejections was recorded in clinical units (n = 1749), while the lowest was observed in the emergency department (n = 365) (Table 1 and Table 2). The association between four units and rejection status was statistically significant, although the effect size was low (Pearson χ^2^, *p* < 0.001; Cramér’s V = 0.066). Examination of adjusted standardized residuals revealed that rejection counts were significantly higher than expected in clinical units (25.3), intensive care units (28.9), and the emergency department (17.9), and significantly lower than expected in outpatient clinics (−47.7).

However, when assessments were based on rejection rates rather than absolute counts, a different pattern emerged. The highest rejection rate was recorded in the emergency department (1.92%), while the lowest was observed in outpatient clinics (0.34%) (Table 1 and Table 2).

### 3.2. Rejection Rates by Time Interval

Rejection rates were found to be non-uniformly distributed across the time intervals (*p* < 0.001). The highest rejection rate was observed during the Z2 interval (04:00–08:00) at 1.98%, while the lowest was recorded during Z3 (08:00–12:00) at 0.45% (Table 1). The association between time interval and rejection status was statistically significant, though the effect size remained low (*p* < 0.001; Cramér’s V = 0.045). Adjusted standardized residuals indicated that rejection counts during Z2 were significantly higher than expected (residual = 28.8), whereas those during Z3 were significantly lower than expected (residual = −22.4).

### 3.3. Rejection Reasons

Analysis of rejection reasons revealed that the majority of rejections were attributable to hemolysis (n = 2073; 47.5%) and clotted specimens (n = 1150; 26.3%) (Table 2). The association between rejection reason and rejection status was statistically significant (*p* < 0.001), with the effect size considerably higher than that observed for the time variable (Cramér’s V = 0.053). Adjusted standardized residuals were markedly elevated for hemolysis (76.8) and clot formation (12.8), and substantially negative for the remaining categories (Insufficient specimen: −13.3; Incorrect collection tube: −21.1; Other: −34.4).

### 3.4. Joint Analysis of Clinical Unit and Time Interval

The combined distribution of rejection counts by clinical unit and time interval was statistically significant (Pearson χ^2^, *p* < 0.001), with a moderate effect size (Cramér’s V = 0.280). The highest rejection count was recorded in clinical unit during Z2 (n = 494), while the highest rejection rate was observed in intensive care units during Z2 (5.13%) (Table 1).

### 3.5. Joint Analysis of Clinical Unit and Rejection Reason

When units and rejection reason were analyzed jointly, the highest rejection count was attributable to hemolysis in clinical units (n = 891), whereas the highest rejection rate was associated with hemolysis-related rejections in the emergency department (2.77%). Regarding the distribution of rejection reasons by unit, rejection count of hemolysis was identified as the predominant error type in clinical units, outpatient clinics, and the emergency department, while clot formation was more prominent in intensive care units (Table 2). The combined distribution was statistically significant (Pearson χ^2^, *p* < 0.001), with a low-to-moderate effect size (Cramér’s V = 0.176). Adjusted standardized residuals indicated that clotted specimens in intensive care units were significantly higher than expected (residual = 17.3), while hemolysis-related rejections exceeded expected values in the emergency department (8.3), outpatient clinics (2.3), and clinical units (3.5).

### 3.6. Three-Way Analysis of Clinical Unit, Time Interval, and Rejection Reason

The three-way analysis revealed that the highest rejection rate in intensive care units occurred during Z2 and was attributable to clot formation (3.58%). In the emergency department, hemolysis-related rejections were elevated during Z3 (3.80%). In clinical units, hemolysis-related rejection rates were particularly pronounced during Z3 (2.42%) and Z4 (2.64%). With the exception of the relatively high hemolysis-related rejection rate observed during the Z1 in outpatient clinics (1.27%), rejection rates across all other error type and time interval combinations in outpatient clinics were markedly lower than those observed in the other units (Table 3).

### 3.7. Negative Binomial Regression Analysis

The negative binomial regression model demonstrated that rejection risk varied significantly according to both units and time interval (*p* < 0.001 for both). With outpatient clinics as the reference category, rejection risk was 3.46-fold higher in clinical units, 4.76-fold higher in intensive care units, and 4.22-fold higher in the emergency department. With respect to time intervals, using Z3 as the reference, only the Z2 interval was found to be independently associated with a significantly increased rejection risk (IRR = 1.63; 95% CI: 1.22–2.19; *p* < 0.001) (Table 4).

**Figure 1:**
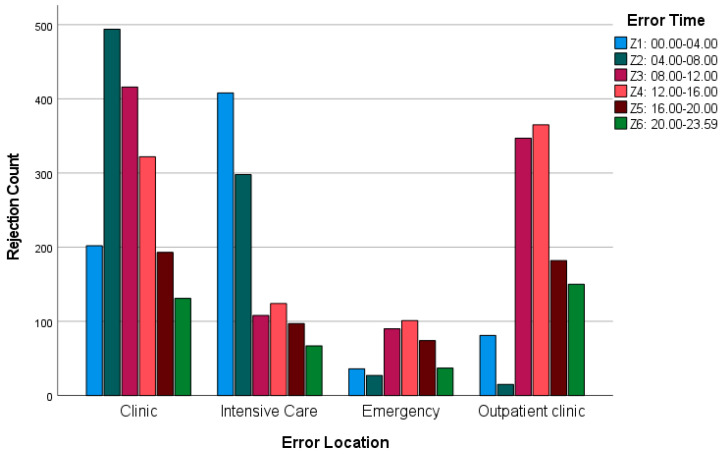
Error Time Intervals on Specimen Rejection Rates.

**Figure 2:**
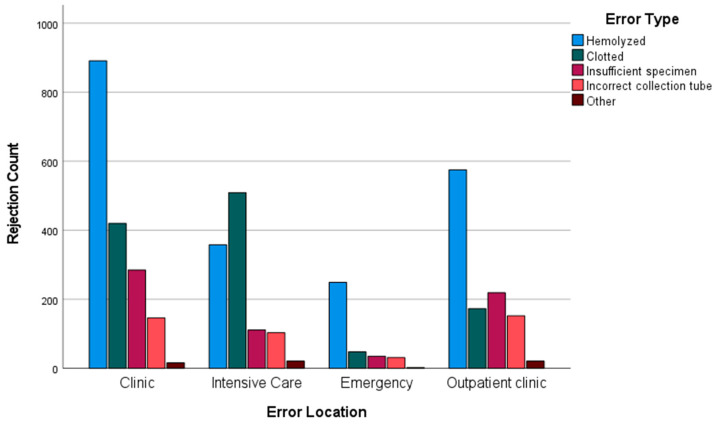
Rejection Count on Specimen Rejection Rates.

When unit–time interval combinations were compared against the reference group (outpatient clinic–Z3), rejection rates differed significantly across combinations (*p* < 0.001). The highest relative risk was identified in intensive care units during Z2 (IRR = 23.86), followed by the emergency department during Z3 (IRR = 11.73), Z4 (IRR = 11.2), and Z5 (IRR = 9.7). In clinical units, the highest relative risks were observed during Z4 (IRR = 9.78), Z2 (IRR = 9.33), and Z3 (IRR = 8.85), while in outpatient clinics, elevated relative risks were noted during Z1 (IRR = 4.99) and Z6 (IRR = 3.14) (Table 5 and Figure 1 and Figure 2).

## 4. Discussion

This study examined preanalytical specimen rejections through a multidimensional analytical framework encompassing unit, time interval, and rejection reason, drawing on a large-scale dataset of 552,450 specimens. To our knowledge, this represents one of the largest single-center analyses conducted in the field of preanalytical quality. The sample sizes reported in previously published studies addressing preanalytical error rates fall considerably short of the volume examined in the present work [1,2,3]. For instance, the landmark study by Plebani and Carraro analyzed 40,490 specimens [7], while the same group’s subsequent investigation extended this to approximately 70,000 specimens [8]. A substantial proportion of the work attributed to Lippi and Simundic consists of systematic reviews or multicenter quality improvement initiatives that draw comparisons across diverse institutional datasets rather than relying on a fixed sample size [1,2,5]. Against this backdrop, the present study—encompassing more than 550,000 specimens—offers a marked advantage in terms of both statistical power and analytical scope relative to the existing literature.

The overall rejection rate of 0.79% observed in this study falls within the 0.3–1.0% range consistently reported across the literature [2,3,14]. Nevertheless, the absolute rejection count of 4365 specimens underscores a critical practical reality: even seemingly low error rates can translate into a substantial operational burden in high-volume laboratory settings [9,13].

When units were evaluated by absolute rejection count, clinical units accounted for the greatest number of rejections. However, rate-based analysis revealed a markedly different picture, with the emergency department recording the highest rejection rate (1.92%), followed by intensive care units (1.75%) and clinical units (1.33%). This distribution reflects the decisive influence of workload intensity, time pressure, and patient management pace on preanalytical process quality, and is consistent with prior studies reporting elevated preanalytical error rates in emergency and critical care environments [3,7,8,15].

The unit-specific distribution of rejection reasons represents an expected yet clinically meaningful finding. Hemolysis emerged as the predominant rejection reason in all unit categories. Although hemolysis has been firmly established as the most frequent preanalytical error in the literature [4,5,6], the detailed unit-level distribution of error types has not been systematically addressed in the majority of prior studies. More recent investigations have demonstrated that hemolysis occurs at disproportionately higher rates in emergency department specimens, attributing this pattern to blood collection under time constraints, intravenous catheter sampling practices, and variability in transport conditions [15,16]. The findings of the present study are consistent with these observations, supporting the notion that workflow characteristics and procedural heterogeneity in emergency and outpatient settings exert a determinative influence on preanalytical quality.

The temporal analysis constitutes one of the most distinctive contributions of this study. To our knowledge, no previous study has systematically monitored specimen rejection rates across six equal four-hour intervals spanning a full 24 h period. Our analyses demonstrated a non-uniform intraday distribution of rejection rates, with the highest rate observed during Z2 and the lowest during Z3. This pattern implies that the relationship between workload and error frequency is not linear—a finding that warrants particular attention given that workload has frequently been cited as a primary driver of preanalytical errors in the literature [5,9], yet its temporal distribution has received comparatively little systematic investigation.

The pronounced operational heterogeneity in sample volume across different time intervals warrants careful contextualization, particularly regarding the critical care setting. Our findings demonstrate that the sample density originating from intensive care units (ICUs) during the Z1 interval (00:00–04:00) was up to two-fold higher than that observed during the subsequent daytime periods (Z2–Z6). This marked temporal skewness is a direct manifestation of established institutional workflows and clinical paradigms inherent to critical care medicine rather than an artifact of sampling bias. In our hospital, routine phlebotomy procedures and biochemical monitoring for critically ill patients are commonly concentrated during the late overnight and early morning hours. This procedural timing guarantees that comprehensive laboratory profiles are fully processed, validated, and integrated into the electronic health records prior to the commencement of morning multidisciplinary patient visit. Consequently, the availability of real-time objective data at the start of the day empowers critical care physicians to make high-stakes, data-driven therapeutic adjustments—such as refining mechanical ventilation parameters, modulating continuous vasoactive infusions, and formulating individualized daily care strategies—during their bedside evaluations. This prominent temporal peak therefore represents a deliberate operational alignment with clinical demands, rather than a systemic distortion within the preanalytical framework itself.

Interaction analysis demonstrated that the association between sampling time and rejection risk was not uniform across clinical departments. To evaluate this relationship, unit × time interaction terms were incorporated into the regression model, and the results are presented in Table 5. Clinical units demonstrated increased rejection risk during the Z2, Z3, and Z4 intervals, potentially reflecting periods of increased workflow intensity and higher specimen volume. In contrast, the ICU exhibited a particularly pronounced interaction effect during the Z2 interval, where rejection risk was substantially greater than that observed in other departments and time periods. This finding may be explained by the concentration of routine monitoring procedures and blood collection activities during early morning hours, combined with the increased complexity of specimen collection in critically ill patients, who frequently present with abnormal coagulation status, vascular access difficulties, and higher clinical acuity. Although ICU personnel are generally experienced, the clustering of high-intensity clinical activities within this time window may further contribute to increased preanalytical vulnerability. Similarly, elevated rejection risk observed in the emergency department during the Z3, Z4, and Z5 intervals may reflect periods of increased patient throughput and operational workload. These findings suggest that preanalytical quality improvement strategies should consider both department-specific workflows and time-dependent operational factors rather than evaluating these variables independently.

Outpatient samples collected during the Z3 interval were selected as the reference category because this group demonstrated the lowest rejection rate during preliminary descriptive analyses. The lower rejection frequency observed in this setting may reflect the influence of standardized phlebotomy practices, dedicated sampling personnel, close proximity between collection areas and laboratory services, and more stable workflow conditions. In contrast, clinical units, intensive care units, and emergency departments are characterized by greater workflow variability, increased patient complexity, and more decentralized specimen collection processes, all of which may increase susceptibility to preanalytical errors.

In this context, the study by Nirwan et al. reported error rates of 1.6% during daytime, 2.0% during evening, and 2.1% during night shifts, suggesting a progressive increase in error risk across successive shift transitions [17]. When interpreted alongside the findings of the present study, these data collectively indicate that preanalytical errors emerge through distinct mechanisms at different times of day. Particularly noteworthy is the observation that rejection rates were at their lowest during Z3, which corresponds to the period of peak operational activity. This counterintuitive finding may be explained by the greater presence of experienced staff during morning hours and the higher degree of process standardization achieved during this period [5,11,18,19].

Specimen collection workflows differ substantially between clinical settings within our institution. In intensive care units, blood sampling is routinely performed by bedside clinical staff across all shifts, whereas outpatient sampling is predominantly conducted by dedicated phlebotomy personnel. These differences in collection practices, staffing models, and workflow organization may partially contribute to department-specific variations in preanalytical error rates.

The identification of Z2 as the sole independently significant risk factor in the negative binomial regression model (IRR = 1.63) provides quantitative evidence that temporal variation is associated with preanalytical specimen rejection risk. Nevertheless, the explanatory contribution of time as an isolated variable appeared limited, whereas the associated risk increased substantially when clinical unit was incorporated into the model. These findings support the multifactorial nature of preanalytical error risk and highlight the importance of evaluating temporal and unit-specific factors in an integrated framework [11]. Negative binomial regression analysis was preferred over Poisson regression due to overdispersion in specimen rejection count data, where variance exceeded the mean values. Time interval and units were included as fixed factors in the regression model. To account for differences in the relevant denominator across error types, the natural logarithm of the total number of corresponding specimen types was included as an offset. Accordingly, the regression models were specified to estimate stratum-level specimen rejection rates.

Among the most meaningful contributions of this study to the existing literature is the demonstration that time may serve as an independent quality indicator for the preanalytical phase. Preanalytical quality has traditionally been operationalized through outcome-based measures such as hemolysis and clot formation rates (9,13), while the temporal variability of these indicators has remained largely overlooked. The findings presented here establish time as a critical parameter that modulates the expression of all such variables, thereby contributing to the closure of a significant gap in the preanalytical quality monitoring literature.

## 5. Limitations

Several limitations should be considered when interpreting the findings of this study. First, the analysis was based exclusively on rejection records extracted from the laboratory information system (LIS). Consequently, the accuracy of the dataset depended on the correctness and completeness of error classification at the time of specimen evaluation. Because individual rejection decisions were made by different laboratory personnel as part of routine practice, the possibility of interobserver variability in the application of rejection criteria cannot be entirely excluded. Furthermore, some preanalytical incidents may not have been formally documented, resulting in potential underreporting of rejection events.

Second, detailed information regarding specimen collection practices at the point of care was unavailable. Variables such as venipuncture technique, collector experience, adherence to phlebotomy guidelines, patient-related factors, transportation conditions before laboratory receipt, and local workflow characteristics within individual clinical units could not be assessed. Similarly, personnel-related variables, including staff qualifications, professional experience, workload, training status, and competency levels, were not available within the dataset and therefore could not be incorporated into the analysis. These unmeasured factors may have contributed to variability in preanalytical error rates. Another important limitation concerns the assessment of hemolysis. The present study evaluated hemolysis only when it resulted in visible specimen rejection during the preanalytical phase. Hemolytic samples identified subsequently during the analytical phase through automated hemolysis detection systems or quantitative HIL (hemolysis–icterus–lipemia) indices were not included in the analysis. Consequently, the reported hemolysis-related rejection rates likely reflect only macroscopically detectable hemolysis and may underestimate the true prevalence of hemolytic interference within the overall testing process.

In addition, rejection criteria for certain specimen quality defects inherently involve a degree of professional judgment. Although standardized laboratory procedures were in place, subjective interpretation of borderline specimens may have introduced minor inconsistencies among personnel responsible for specimen acceptance and rejection decisions.

The study was also conducted during a period of relative operational stability within the laboratory. No major automation upgrades, organizational restructuring, or substantial workflow modifications occurred during the study interval. While this reduced the likelihood of confounding effects related to system transitions, it limits the applicability of the findings to periods characterized by significant technological implementation, staffing changes, or organizational transformation. Future longitudinal studies encompassing such transition periods may provide a more comprehensive understanding of how operational changes influence preanalytical performance. Although the dataset covered an extended observation period, the primary objective of the study was to investigate temporal variation in rejection patterns according to sampling time intervals. Therefore, potentially relevant factors such as seasonal variation, fluctuations in laboratory workload, and long-term staffing dynamics were not systematically examined. Likewise, while weekday and weekend comparisons were explored, differences in sample distribution between these periods may have influenced the robustness of subgroup analyses. Future multicenter investigations incorporating prospective data collection, detailed staffing metrics, comprehensive specimen collection variables, and analyte-specific rejection tracking may further clarify the complex determinants of preanalytical laboratory errors.

## Figures and Tables

**Table 1:** Number and rate of rejections by unit and time period.

	Time Interval	Z1	Z2	Z3	Z4	Z5	Z6	Total
Unit								
**Clinical units**	Rejection counts	202	494	416	322	193	131	1758
	Number of Samples	33,335	26,847	27,408	18,296	14,194	12,353	132,433
	Rejection Rate (%)	**0.61**	**1.84**	**1.52**	**1.76**	**1.36**	**1.06**	**1.33**
**Intensive Care**	Rejection counts	408	298	108	124	97	67	1102
	Number of Samples	31,735	5804	6461	7598	6359	4983	62,940
	Rejection Rate (%)	**1.29**	**5.13**	**1.67**	**1.63**	**1.53**	**1.34**	**1.75**
**Emergency**	Rejection counts	36	27	90	101	74	37	365
	Number of Samples	2450	2167	3515	4168	3467	3260	19,027
	Rejection Rate (%)	**1.47**	**1.25**	**2.56**	**2.42**	**2.13**	**1.13**	**1.92**
**Outpatient Clinic**	Rejection counts	81	15	347	365	182	150	1140
	Number of Samples	7105	7198	175,120	92,841	34,279	21,507	338,050
	Rejection Rate (%)	**1.14**	**0.21**	**0.20**	**0.39**	**0.53**	**0.70**	**0.34**
**Total**	Rejection counts	727	834	961	912	546	385	4365
	Number of Samples	74,625	42,016	212,504	122,903	58,299	42,103	552,450
	Rejection Rate (%)	**0.97**	**1.98**	**0.45**	**0.74**	**0.94**	**0.91**	**0.79**

**Table 2:** Number and rate of rejections by unit and reason for rejection.

	Rejection Reason	Hemolyzed	Clotted	Insufficient Specimen	Incorrect Collection Tube	Other	Total
Unit							
**Clinical units**	Rejection counts	891	420	285	146	16	1758
	Number of Samples	53,657	111,522	132,433	132,433	132,433	132,433
	(Number of Sample Type)	(38,645 ^a^ + 15,012 ^b^)	(38,645 ^a^ + 72,877 ^c^)	(38,645 ^a^ + 15,012 ^b^ + 72,877 ^c^ + 5899 ^d^)	(38,645 ^a^ + 15,012 ^b^ + 72,877 ^c^ + 5899 ^d^)	(38,645 ^a^ + 15,012 ^b^ + 72,877 ^c^ + 5899 ^d^)	(38,645 ^a^ + 15,012 ^b^ + 72,877 ^c^ + 5899 ^d^)
	Rejection Rate (%)	**1.66**	**0.38**	**0.22**	**0.11**	**0.01**	**1.33**
**Intensive Care**	Rejection counts	358	509	111	103	21	1102
	Number of Samples	26,415	47,224	62,940	62,940	62,940	62,940
	* (Number of Sample Type)	(16,084 ^a^ + 10,331 ^b^)	(16,084 ^a^ + 31,140 ^c^)	(16,084 ^a^ + 10,331 ^b^ + 31,140 ^c^ + 5385 ^d^)	(16,084 ^a^ + 10,331 ^b^ + 31,140 ^c^ + 5385 ^d^)	(16,084 ^a^ + 10,331 ^b^ + 31,140 ^c^ + 5385 ^d^)	(16,084 ^a^ + 10,331 ^b^ + 31,140 ^c^ + 5385 ^d^)
	Rejection Rate (%)	**1.36**	**1.08**	**0.18**	**0.16**	**0.03**	**1.75**
**Emergency**	Rejection counts	249	48	35	31	2	365
	Number of Samples	8979	17,068	19,027	19,027	19,027	19,027
	* (Number of Sample Type)	(8135 ^a^ + 844 ^b^)	(8135 ^a^ + 8933 ^c^)	(8135 ^a^ + 844 ^b^ + 8933 ^c^ + 1115 ^d^)	(8135 ^a^ + 844 ^b^ + 8933 ^c^ + 1115 ^d^)	(8135 ^a^ + 844 ^b^ + 8933 ^c^ + 1115 ^d^)	(8135 ^a^ + 844 ^b^ + 8933 ^c^ + 1115 ^d^)
	Rejection Rate (%)	**2.77**	**0.28**	**0.18**	**0.16**	**0.01**	**1.92**
**Outpatient Clinic**	Rejection counts	575	173	219	152	21	1140
	Number of Samples	172,319	268,470	338,050	338,050	338,050	338,050
	* (Number of Sample Type)	(137,373 ^a^ + 34,946 ^b^)	(137,373 ^a^ + 131,097 ^c^)	(137,373 ^a^ + 34,946 ^b^ + 131,097 ^c^ + 34,634 ^d^)	(137,373 ^a^ + 34,946 ^b^ + 131,097 ^c^ + 34,634 ^d^)	(137,373 ^a^ + 34,946 ^b^ + 131,097 ^c^ + 34,634 ^d^)	(137,373 ^a^ + 34,946 ^b^ + 131,097 ^c^ + 34,634 ^d^)
	Rejection Rate (%)	**0.33**	**0.06**	**0.06**	**0.04**	**0.01**	**0.34**
**Total**	Rejection counts	2073	1150	650	432	60	4365
	Number of Samples	261,370	444,284	552,450	552,450	552,450	552,450
	* (Number of Sample Type)	(200,237 ^a^ + 61,133 ^b^)	(200,237 ^a^ + 244,047 ^c^)	(200,237 ^a^ + 61,133 ^b^ + 244,047 ^c^ + 47,033 ^d^)	(200,237 ^a^ + 61,133 ^b^ + 244,047 ^c^ + 47,033 ^d^)	(200,237 ^a^ + 61,133 ^b^ + 244,047 ^c^ + 47,033 ^d^)	(200,237 ^a^ + 61,133 ^b^ + 244,047 ^c^ + 47,033 ^d^)
	Rejection Rate (%)	**0.79**	**0.26**	**0.12**	**0.08**	**0.01**	**0.79**

* (Number of Sample Type): Specimen types associated with observed rejection reasons. ^a^: Serum, ^b^: Plasma, ^c^: Whole Blood Sample, ^d^: Urine.

**Table 3:** Number and rate of rejections by unit, cause of error, and time period.

		Time Interval	Z1	Z2	Z3	Z4	Z5	Z6	Total
Unit	Error Type								
**Clinical units**	**Hemolyzed**	Rejection counts	116	147	309	184	79	56	891
		Number of Samples	15,296	10,139	12,774	6958	4548	3942	53,657
		Rejection Rate (%)	**0.76**	**1.45**	**2.42**	**2.64**	**1.74**	**1.42**	**1.66**
	**Clotted**	Rejection counts	50	242	20	27	42	39	420
		Number of Samples	29,397	24,184	21,156	14,530	11,940	10,315	111,522
		Rejection Rate (%)	**0.17**	**1.00**	**0.09**	**0.19**	**0.35**	**0.38**	**0.38**
	**Insufficient** **specimen**	Rejection counts	24	57	61	71	53	19	285
		Number of Samples	33,335	26,847	27,408	18,296	14,194	12,353	132,433
		Rejection Rate (%)	**0.07**	**0.21**	**0.22**	**0.39**	**0.37**	**0.15**	**0.22**
	**Incorrect** **collection** **tube**	Rejection counts	9	42	25	37	17	16	146
		Number of Samples	33,335	26,847	27,408	18,296	14,194	12,353	132,433
		Rejection Rate (%)	**0.03**	**0.16**	**0.09**	**0.20**	**0.12**	**0.13**	**0.11**
	**Other**	Rejection counts	3	6	1	3	2	1	16
		Number of Samples	33,335	26,847	27,408	18,296	14,194	12,353	132,433
		Rejection Rate (%)	**0.01**	**0.02**	**0.00**	**0.02**	**0.01**	**0.01**	**0.01**
	**Total**	Rejection counts	202	494	416	322	193	131	1758
		Number of Samples	33,335	26,847	27,408	18,296	14,194	12,353	132,433
		Rejection Rate (%)	**0.61**	**1.84**	**1.52**	**1.76**	**1.36**	**1.06**	**1.33**
**Intensive Care**	**Hemolyzed**	Rejection counts	174	79	36	34	24	11	358
		Number of Samples	18,868	2404	1312	1398	1114	1319	26,415
		Rejection Rate (%)	**0.92**	**3.29**	**2.74**	**2.43**	**2.15**	**0.83**	**1.36**
	**Clotted**	Rejection counts	187	177	26	28	51	40	509
		Number of Samples	22,673	4947	5184	5930	4822	3668	47,224
		Rejection Rate (%)	**0.82**	**3.58**	**0.50**	**0.47**	**1.06**	**1.09**	**1.08**
	**Insufficient** **specimen**	Rejection counts	15	17	18	37	17	7	111
		Number of Samples	31,735	5804	6461	7598	6359	4983	62,940
		Rejection Rate (%)	**0.05**	**0.29**	**0.28**	**0.49**	**0.27**	**0.14**	**0.18**
	**Incorrect** **collection** **tube**	Rejection counts	31	21	22	21	2	6	103
		Number of Samples	31,735	5804	6461	7598	6359	4983	62,940
		Rejection Rate (%)	**0.10**	**0.36**	**0.34**	**0.28**	**0.03**	**0.12**	**0.16**
	**Other**	Rejection counts	1	4	6	4	3	3	21
		Number of Samples	31,735	5804	6461	7598	6359	4983	62,940
		Rejection Rate (%)	**0.00**	**0.07**	**0.09**	**0.05**	**0.05**	**0.06**	**0.03**
	**Total**	Rejection counts	408	298	108	124	97	67	1102
		Number of Samples	31,735	5804	6461	7598	6359	4983	62,940
		Rejection Rate (%)	**1.29**	**5.13**	**1.67**	**1.63**	**1.53**	**1.34**	**1.75**
**Emergency**	**Hemolyzed**	Rejection counts	23	18	61	70	52	25	249
		Number of Samples	1204	1015	1605	1968	1639	1548	8979
		Rejection Rate (%)	**1.91**	**1.77**	**3.80**	**3.56**	**3.17**	**1.61**	**2.77**
	**Clotted**	Rejection counts	10	5	5	7	14	7	48
		Number of Samples	2263	1969	3057	3678	3120	2981	17,068
		Rejection Rate (%)	**0.44**	**0.25**	**0.16**	**0.19**	**0.45**	**0.23**	**0.28**
	**Insufficient** **specimen**	Rejection counts	3	2	13	9	6	2	35
		Number of Samples	2450	2167	3515	4168	3467	3260	19,027
		Rejection Rate (%)	**0.12**	**0.09**	**0.37**	**0.22**	**0.17**	**0.06**	**0.18**
	**Incorrect** **collection** **tube**	Rejection counts	0	2	11	13	2	3	31
		Number of Samples	2450	2167	3515	4168	3467	3260	19,027
		Rejection Rate (%)	**0.00**	**0.09**	**0.31**	**0.31**	**0.06**	**0.09**	**0.16**
	**Other**	Rejection counts	0	0	0	2	0	0	2
		Number of Samples	2450	2167	3515	4168	3467	3260	19,027
		Rejection Rate (%)	**0.00**	**0.00**	**0.00**	**0.05**	**0.00**	**0.00**	**0.01**
	**Total**	Rejection counts	36	27	90	101	74	37	365
		Number of Samples	2450	2167	3515	4168	3467	3260	19,027
		Rejection Rate (%)	**1.47**	**1.25**	**2.56**	**2.42**	**2.13**	**1.13**	**1.92**
**Outpatient Clinic**	**Hemolyzed**	Rejection counts	44	8	180	186	92	65	575
		Number of Samples	3458	3692	88,300	48,863	17,411	10,595	172,319
		Rejection Rate (%)	**1.27**	**0.22**	**0.20**	**0.38**	**0.53**	**0.61**	**0.33**
	**Clotted**	Rejection counts	24	6	42	32	29	40	173
		Number of Samples	6238	5356	136,049	74,239	27,899	18,689	268,470
		Rejection Rate (%)	**0.38**	**0.11**	**0.03**	**0.04**	**0.10**	**0.21**	**0.06**
	**Insufficient** **specimen**	Rejection counts	9	0	69	77	36	28	219
		Number of Samples	7105	7198	175,120	92,841	34,279	21,507	338,050
		Rejection Rate (%)	**0.13**	**0.00**	**0.04**	**0.08**	**0.11**	**0.13**	**0.06**
	**Incorrect** **collection** **tube**	Rejection counts	4	1	49	64	20	14	152
		Number of Samples	7105	7198	175,120	92,841	34,279	21,507	338,050
		Rejection Rate (%)	**0.06**	**0.01**	**0.03**	**0.07**	**0.06**	**0.07**	**0.04**
	**Other**	Rejection counts	0	0	7	6	5	3	21
		Number of Samples	7105	7198	175,120	92,841	34,279	21,507	338,050
		Rejection Rate (%)	**0.00**	**0.00**	**0.00**	**0.01**	**0.01**	**0.01**	**0.01**
	**Total**	Rejection counts	81	15	347	365	182	150	1140
		Number of Samples	7105	7198	175,120	92,841	34,279	21,507	338,050
		Rejection Rate (%)	**1.14**	**0.21**	**0.20**	**0.39**	**0.53**	**0.70**	**0.34**

**Table 4:** Main Effects of Units and Error Time Intervals on Specimen Rejection Rates.

Variable	Category	IRR	%95 CI	*p*
**Unit**	Clinical	3.46	2.76–4.32	<0.001
	Intensive care	4.76	3.83–5.92	<0.001
	Emergency	4.22	3.29–5.42	<0.001
	Outpatient Clinic	1	Reference	—
**Time**	Z1	0.77	0.57–1.05	0.103
	Z2	1.63	1.22–2.19	<0.001
	Z3	1	Reference	—
	Z4	1.25	0.94–1.66	0.127
	Z5	1.14	0.86–1.50	0.366
	Z6	0.98	0.73–1.31	0.896

IRR: incidence rate ratio; values were estimated using negative binomial regression with a log link and an offset for the natural logarithm of the total number of specimens. IRR > 1 indicates a higher specimen rejection rate, whereas IRR < 1 indicates a lower specimen rejection rate compared with the reference category.

**Table 5:** Interaction of Units and Error Time Intervals on Specimen Rejection Rates.

Unit	Time	IRR	%95 CI	*p*
**Clinical**	Z1	2.97	1.86–4.73	<0.001
	Z2	9.33	6.29–13.83	<0.0001
	Z3	8.85	5.36–14.06	<0.0001
	Z4	9.78	6.11–15.67	<0.0001
	Z5	6.59	4.27–10.18	<0.0001
	Z6	5.05	3.21–7.94	<0.001
**Intensive care**	Z1	6.4	4.24–9.66	<0.0001
	Z2	23.86	15.51–36.71	<0.0001
	Z3	8.02	5.02–12.81	<0.0001
	Z4	7.82	5.07–12.06	<0.0001
	Z5	7.37	4.48–12.13	<0.001
	Z6	6.14	3.78–9.99	<0.001
**Emergency**	Z1	6.22	3.53–10.94	<0.001
	Z2	5.2	3.02–8.93	<0.001
	Z3	11.73	6.88–20.02	<0.0001
	Z4	11.2	6.75–18.58	<0.0001
	Z5	9.7	5.84–16.10	<0.0001
	Z6	4.83	2.69–8.68	<0.001
**Outpatient Clinic**	Z1	4.99	3.00–8.28	<0.001
	Z2	0.88	0.45–1.72	0.716
	Z3	1	Reference	—
	Z4	1.96	1.29–2.98	0.002
	Z5	2.48	1.63–3.79	<0.001
	Z6	3.14	2.04–4.84	<0.001

IRR: incidence rate ratio; values were estimated using negative binomial regression with a log link and an offset for the natural logarithm of the total number of specimens. IRR > 1 indicates a higher specimen rejection rate, whereas IRR < 1 indicates a lower specimen rejection rate compared with the reference category.

## Data Availability

The data presented in this study are available from the corresponding author upon reasonable request, subject to institutional and ethical regulations.
